# La gangrène ischémique de la verge chez un patient diabétique: à propos d'un cas

**DOI:** 10.11604/pamj.2015.21.74.6906

**Published:** 2015-05-29

**Authors:** Tarik El Moutawakkil, Khalid Elmortaji, Amine Arsalane, Driss Ellahik, Rachid Aboutaieb, Redouane Rabii, Fethi Meziane

**Affiliations:** 1 Service d'Urologie, CHU Ibn Rochd, Casablanca, Maroc

**Keywords:** Diabète, gangrène ischémique, verge, diabetes, ischemic gangrene, penis

## Abstract

La gangrène de la verge est une pathologie rare mais grave. Les étiologies sont diverses avec essentiellement le diabète. Nous rapportons le cas d'un patient de 58 ans, diabétique et tabagique chronique ayant présenté une nécrose de la verge et avait bénéficié d'une amputation totale après deux interventions chirurgicale. L’évolution a été marquée par l'installation d'un sepsis et décès du patient.

## Introduction

La gangrène de la verge est une entité rare qui témoigne souvent d'une artériopathie périphérique sévère et peut poser des difficultés dans la prise en charge. Elle survient essentiellement chez les patients diabétiques, artéritiques ou insuffisants rénaux chroniques. Bien que le pénis et le gland distal aient une abondante vascularisation artérielle, l′occlusion artérielle peut provoquer des nécroses distales semblables à la gangrène ischémique souvent notée dans les extrémités des membres. L'imagerie médicale essentiellement l'IRM, permet de bien définir les limites de la zone nécrosée. Le traitement est essentiellement chirurgical. Nous rapportons un cas de nécrose de la verge, traité par amputation totale.

## Patient et observation

**Figure 1:**
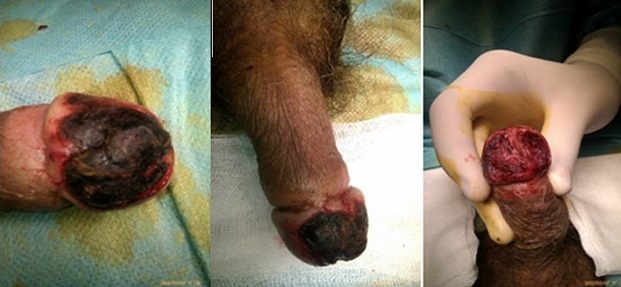
Nécrose localisée au niveau de la face dorsale du gland

Un homme de 58 ans, tabagique chronique à 22 paquet-année, diabète type 2, découvert il y a 5 ans, sous antidiabétique oraux, mal équilibré. Il s'est présenté aux urgences pour coloration noirâtre du gland apparue une semaine auparavant. A l'examen clinique, on retrouve un patient ayant un retard mental, stable sur le plan hémodynamique, apyrétique. L'examen des organes génitaux externes met en évidence une nécrose localisée au niveau de la face dorsale du gland (Figure 1). Les bourses d'aspect normales. Pas de signes urinaires. Labstix: glycosurie sans cétonurie. Le bilan biologique a montré une hyperglycémie à 2,3 g/l, une hyperleucocytose à 11300/ml, une créatinine à 15 mg/l. L'ECBU stérile. L'IRM n’était pas disponible dans l'immédiat. Le patient fut hospitalisé et mis sous antibiotiques et héparine avec équilibration du diabète par insulinothérapie.

**Figure 2:**
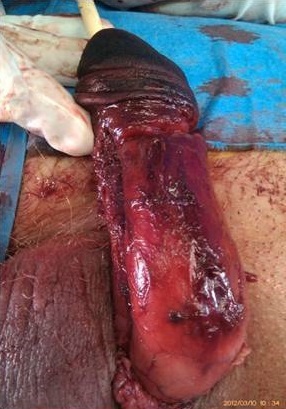
Extension de la nécrose sur toute la longueur de la verge

**Figure 3:**
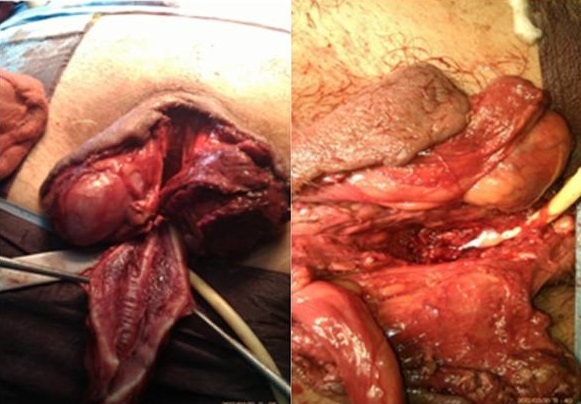
Images en per-opératoire d'amputation de la verge

Il a bénéficié en urgence d'une nécrosectomie limitée au niveau du gland, suivi de soins locaux. L’évolution s'est marquée par la détérioration de l’état général, une acido-cétose diabétique et extension de la nécrose sur toute la longueur de la verge (Figure 2). Le patient fut transféré en réanimation. Une reprise chirurgicale avec amputation de la verge a été réalisé (Figure 3). L’évolution post-opératoire est marquée par l'installation d'un sepsis. Le patient est décédé trois jours après.

## Discussion

La gangrène du pénis constitue une complication rare mais grave. Seulement quelques cas de gangrène du pénis ont été rapportés dans la littérature. Elle témoigne souvent d'une artériopathie périphérique sévère [1–4]. L'ischémie de la verge est d'installation progressive [5], car la verge est bien vascularisée par des artères profondes, branches de l'artère honteuse interne: deux artères caverneuses, l'artère bulbaire et l'artère urétrale et par des artères superficielles, branches de l'honteuse externe [6].

Les principales étiologies incriminées sont le diabète sucré et l′accumulation de dépôts de calcium due à l'insuffisance rénale chronique terminale [6]. D'autres causes ont été rapportées [1], notamment l'effet de garrot, les maladies thrombo-emboliques, les troubles de coagulation secondaire à une maladie néoplasique, les traumatismes et l'infection. Dans notre cas, l'artériopathie diabétique serait probablement le facteur incriminé dans la survenue de la gangrène.

La différenciation clinique entre la gangrène sèche due à une maladie ischémique et la gangrène humide avec l′infection est décisive pour le choix du traitement approprié [3]. En cas de gangrène sèche, les deux stratégies de traitement comprennent un traitement conservateur, souvent associé à une circoncision, et pénectomie partielle ou totale [3]. L′indication du traitement conservateur implique généralement de petites lésions circonscrites ou patients à haut risque. Toutefois, un traitement conservateur est souvent suivi par une intervention chirurgicale pour progression de la maladie ou de liquéfaction et le développement de l′infection. La prise en charge expectante devrait être réservée aux patients en état critique ou malades en phase terminale. Il a été recommandé que la circoncision soit pratiquée dans des cas de traitement conservateur pour faciliter l′observation et permettre la cicatrisation sèche [3]. Un traitement agressif est recommandé pour les patients n'ayant pas de comorbidités sévères [7]. Un garrot ne doit pas être utilisé lors de la chirurgie dans ces cas afin d′éviter des dommages supplémentaires aux tissus ischémiques. Harris et Mydlo ont rapporté l'absence de progression de la nécrose chez trois patients ayant bénéficié d'un débridement initial de la nécrose, associé à des soins locaux et antibiothérapie [2]. Stein et al. ont rapporté un taux de mortalité de 71% dans une série de sept patients [4, 5]. Parmi les cinq patients gardés en observation, deux ont eu une résolution spontanée de la gangrène et trois cas une stabilisation. Les deux patients traités par pénectomie et trois des cinq patients gardés en observation sont décédés dans les trois mois suivants. Leur décès n’était pas lié à la gangrène. Les auteurs ont conclu sur l'absence d'avantage de la chirurgie radicale comparée à la surveillance seule. Weiner et Lowe, dans une série de sept patients présentant une gangrène ischémique de la verge au cours du diabète, ont rapporté un taux de mortalité de 57% dans les six mois suivant le diagnostic [2, 5]. Ils ont noté que la pénectomie précoce, même si elle ne diminue pas le taux de mortalité, peut améliorer la qualité de vie en prévenant ou en limitant les complications locorégionales. Avec une sélection appropriée des patients, une intervention chirurgicale peut réussir et offrir une meilleure qualité de vie. Puisque la pénectomie partielle est techniquement plus facile et de conséquences psychologiques moindres que la pénectomie totale, elle est usuellement pratiquée. Dans notre cas, le traitement conservateur a été complété par une pénectomie totale.

## Conclusion

La gangrène sèche de la verge est une entité rare qui témoigne souvent d'une artériopathie périphérique sévère. Le diabète sucré et l'insuffisance rénale terminale sont les principaux facteurs incriminés. Deux stratégies thérapeutiques peuvent être envisagées. Le traitement conservateur et le traitement chirurgical radical basé sur la pénectomie. Pour éviter les complications médico-légales, un consentement éclairé doit être signé, incluant la possibilité de pénectomie, car les signes cliniques peuvent ne pas refléter les constatations per-opératoires.
